# Dynamic hydrogen peroxide levels reveal a rate-dependent sensitivity in B-cell lymphoma signaling

**DOI:** 10.1038/s41598-024-54871-7

**Published:** 2024-02-21

**Authors:** Melde Witmond, Emma Keizer, Bas Kiffen, Wilhelm T. S. Huck, Jessie A. G. L. van Buggenum

**Affiliations:** 1 Institute for Molecules and Materials (IMM), Radboud University Nijmegen, Nijmegen, The Netherlands; 2 Single Cell Discoveries (SCD), Utrecht, The Netherlands

**Keywords:** Biophysical chemistry, Networks and systems biology, Signal transduction, Biochemical networks, Nonlinear dynamics

## Abstract

Although in vivo extracellular microenvironments are dynamic, most in vitro studies are conducted under static conditions. Here, we exposed diffuse large B-cell lymphoma (DLBCL) cells to gradient increases in the concentration of hydrogen peroxide (H_2_O_2_), thereby capturing some of the dynamics of the tumour microenvironment. Subsequently, we measured the phosphorylation response of B-cell receptor (BCR) signalling proteins CD79a, SYK and PLCγ2 at a high temporal resolution via single-cell phospho-specific flow cytometry. We demonstrated that the cells respond bimodally to static extracellular H_2_O_2_, where the percentage of cells that respond is mainly determined by the concentration. Computational analysis revealed that the bimodality results from a combination of a steep dose–response relationship and cell-to-cell variability in the response threshold. Dynamic gradient inputs of varying durations indicated that the H_2_O_2_ concentration is not the only determinant of the signalling response, as cells exposed to more shallow gradients respond at lower H_2_O_2_ levels. A minimal model of the proximal BCR network qualitatively reproduced the experimental findings and uncovered a rate-dependent sensitivity to H_2_O_2_, where a lower rate of increase correlates to a higher sensitivity. These findings will bring us closer to understanding how cells process information from their complex and dynamic in vivo environments.

## Introduction

An important factor in disease development is the extracellular microenvironment of cells. In vivo, the microenvironment of cells is highly dynamic, for instance due to the production or release of small molecules. However, most in vitro research on cellular signalling studies the effects of sudden increases in stimuli (static step stimulations). Although such studies have been very informative, static in vitro study conditions do not reflect the complex and dynamic in vivo environments of cells. Various recent studies show that temporal dynamic stimuli can influence cellular signal processing, decision making, and function^1–6^. A study on human embryonic stem cells elucidated that the SMAD4 protein responds dose-dependently to one morphogen, but in a rate-dependent manner to another morphogen^1^. Johnson et al*.* (2021) discovered both a dose and a rate threshold mechanism that together govern MAPK stress signalling in yeast^2^. The same group showed that human cells survive gradual but not acute osmotic stress, where stress signalling pathways were not activated upon dynamic input patterns^6^. Furthermore, a study on fibroblast cells demonstrated that the NFkB signalling pathway responds to absolute differences in cytokine concentrations, also under dynamic cytokine conditions^4^. O’Donoghue et al*.* (2021) studied T-cells and their capability to selectively filter out oscillatory signals at the minute timescale^3^. Thus, research has revealed a large variety of mechanisms with which different cell systems process dynamic inputs from their environment.

**Figure 1:**
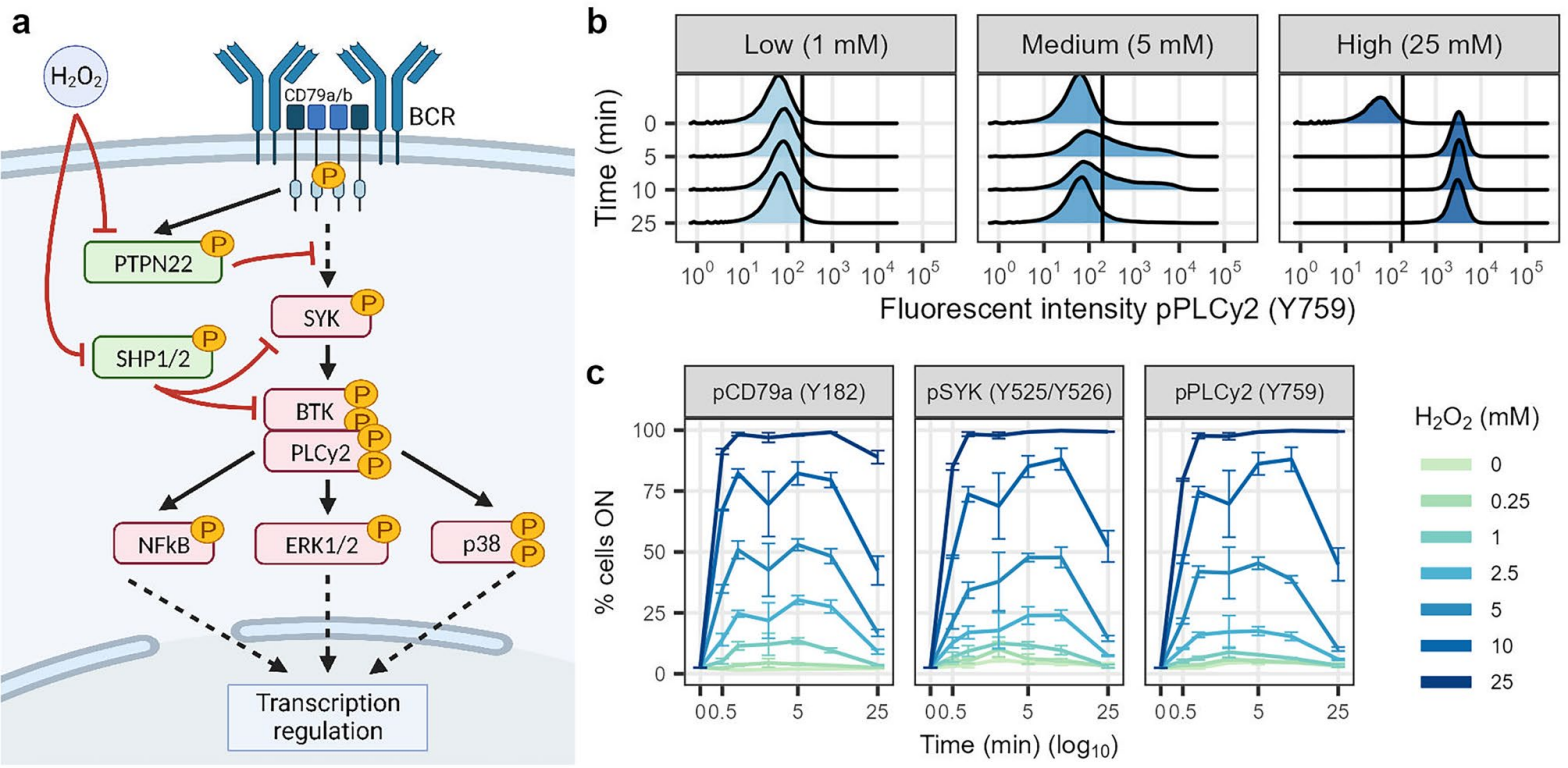
Static H_2_O_2_ exposure results in heterogeneous and dose-dependent phosphorylation of BCR network proteins. (**a**) Schematic of the BCR network, with phosphatases and H_2_O_2_ influence. Kinases are indicated in red, phosphatases in green, sharp black arrows are activating effects, flat red arrows are inhibitory effects. (**b**) Density plots of PLCγ2 phosphorylation response to low (1 mM), medium (5 mM) or high (25 mM) static H_2_O_2_ doses after 0, 5, 10 or 25 min of exposure. Vertical lines indicate the ON/OFF threshold (see Methods for details). (**c**) pCD79a, pSYK and pPLCγ2 response over time (in percentage of cells responding) for different concentrations of H_2_O_2_ (average of 3 replicates per condition with standard deviation as error bars).

One cell type for which it is particularly important to respond to dynamic environments is the B-cell, as these immune cells have to recognise pathogens and process the information to initiate immune responses. However, B-cells have not yet been studied in in vitro dynamic environments. In addition, reactive oxygen species (ROS), such as hydrogen peroxide (H_2_O_2_), play a critical role in healthy B-cell function and lymphoma development^7–9^. H_2_O_2_ in the microenvironment of lymphoma cells can reduce the activation of macrophages and natural killer cells, thereby promoting tumour cell survival^10,11^. Moreover, H_2_O_2_ can cross the cell membrane and alter intracellular signalling by inhibiting phosphatases, which are essential negative signalling regulators, thus promoting proliferation^12^. H_2_O_2_ can activate the B-cell receptor (BCR) network, a major signalling network in lymphoma cells (Fig. 1a). Normally, this network is activated via antigen recognition by the receptor^13,14^. Antigen binding causes the BCR complex, consisting of Immunoglobulin (Ig) and the CD79a/b heterodimer, to cluster and associate with LYN. LYN phosphorylates CD79a/b, which subsequently phosphorylates tyrosine kinase SYK. SYK is a key kinase in the BCR network, able to activate the BTK/PLCγ2 signalling hub, the PI3K/AKT pathway (protein synthesis), and the p38 MAPK pathway (transcription regulation). The BTK/PLCγ2 hub, in turn, can activate the ERK1/2, NFkB and NFAT pathways (transcription regulation). In addition, BCR-proximal phosphatases such as SHP1 and SHP2, PTPN22, SHIP and PTEN are activated through LYN, PI3K, and localised ROS production^15,16^. These phosphatases are essential negative regulators of BCR signalling. H_2_O_2_-induced activation of the BCR signalling is distinctly different from antigen-based activation. H_2_O_2_ oxidises cysteine residues in the active site of the phosphatases, thereby rendering them unable to inhibit the kinases in the network^17^, resulting in active BCR signalling. Indeed, previous research has demonstrated that both healthy primary B-cells and chronic lymphocytic leukemia (CLL) cells respond in vitro to H_2_O_2_ via BCR signalling^8,18–20^.

Since environment sensing is essential for B-cell function but B-cell responses to dynamic conditions have not yet been studied, we have opted to use a diffuse large B-cell lymphoma (DLBCL) cell model and study their signaling response to dynamic H_2_O_2_ concentrations. As a step towards complex dynamic environments, we applied temporal gradients of increasing stimulus to study how DLBCL cells process dynamic inputs and measured signalling responses with single-cell phospho-specific flow cytometry. We demonstrated that the activated B-cell (ABC) type DLBCL cell line HBL1 responds to extracellular H_2_O_2_ by heterogeneously activating various components of the BCR network. Static step stimulations indicated a concentration-dependent signalling response to extracellular H_2_O_2_. However, dynamic H_2_O_2_ input patterns revealed a rate-dependent sensitivity to H_2_O_2_; more shallow gradients induced a response at lower H_2_O_2_ levels. This behaviour was qualitatively reproduced with a mathematical minimal model of the proximal BCR signalling network, suggesting that the rate-dependent sensitivity is inherent to the BCR network.

## Results and discussion

### Static H_2_O_2_ exposure results in bimodal and dose-dependent phosphorylation of BCR network proteins

First, we set out to establish the BCR signalling response to static ROS-induced phosphatase inhibition conditions. The cell model responds to static extracellular H_2_O_2_ by increasing the phosphorylation of upstream BCR proteins CD79a (Y182), SYK (Y525/Y526) and PLCγ2 (Y759) (Fig. 1b,c). The H_2_O_2_ concentrations used here compare to previous work in different B-cell types^18–20^. Interestingly, the single-cell data reveals a heterogeneous response to static concentrations of H_2_O_2_. Low, medium, and high concentrations induce distinct population-level phosphorylation distributions (Fig. 1b [pPLCγ2], Supplementary Fig. S1 [pCD79a + pSYK]). A low dose results in a unimodal OFF response, a medium dose leads to heterogeneous (bimodal) behaviour with a fraction of the cells ON, and a high dose results in a unimodal ON response (see Methods for ON/OFF threshold details). Thus, the cells respond in a digital rather than graded manner to H_2_O_2_, with the proportions between the two subpopulations varying upon input conditions. Such heterogeneous or bimodal population responses upon static H_2_O_2_ stimulation have also been observed in another lymphoma type (CLL)^19,20^, but not in healthy primary B-cells^18,19,21^. The bimodal response to H_2_O_2_ highlights the importance of single-cell data and analysis approaches in signalling studies. A graded response, where all cells respond similarly, can be studied with bulk methods. A digital response with two subpopulations, on the other hand, requires single-cell methods since average values would obscure the heterogeneity in the population.

The percentage of activated cells over time indicates that the response of CD79a, SYK and PLCγ2 is governed by the H_2_O_2_ concentration (Fig. 1c). Signaling activation requires exceeding a dose threshold that lies somewhere between 2.5 and 10 mM H_2_O_2_, depending on the cell-to-cell variability. In addition, the effect of static H_2_O_2_ is transient, likely due to clearance of H_2_O_2_ by proteins and antioxidants in the cell^9^. For higher H_2_O_2_ doses, it takes longer for the system to revert to basal levels. In short, static extracellular H_2_O_2_ induces heterogeneous and dose-dependent activation of the BCR signalling pathway.

**Figure 2:**
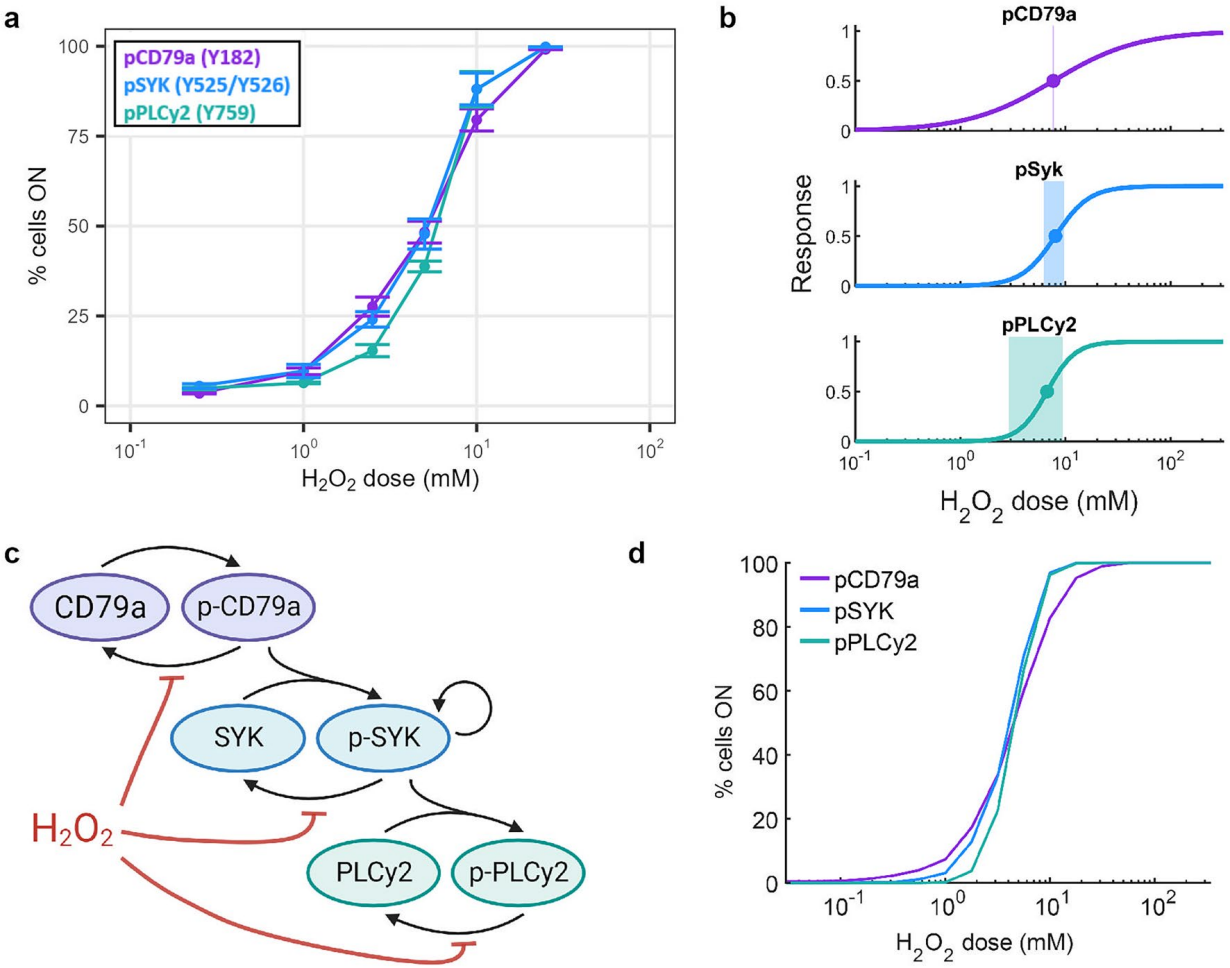
Bimodality results from a combination of a steep dose–response relationship and cell-to-cell variability. (**a**) Experimental dose–response curves of pCD79a, pSYK and pPLCγ2 (t = 10 min). (**b**) Normalised experimental dose–response curves with ${{\text{EC}}}_{50}$ (point) and estimated bimodality range (shaded area; proxy for cell-to-cell variability). (**c**) Minimal model of the BCR network. (**d**) Simulated dose–response curves of pCD79a, pSYK and pPLCγ2.

To gain a better understanding of the origin of the bimodal responses to H_2_O_2_, we created dose–response (DR) curves for each protein from the static stimulation data (t = 10 min; Fig. 2a). The DR of the system is assumed to follow Hill-type kinetics, where the response is characterised by the half-maximal response threshold, ${EC}_{50}$, describing the stimulus level for which the response is half of the maximum, and the Hill coefficient, $H$, determining the steepness of the DR curve (see “Methods”/Eq. (3) for more details). DR curves fitted on the percentage of cells ON give average ${EC}_{50}$ values of 8.9 mM for pCD79a, 7.6 mM for pSYK, and 6.7 mM H_2_O_2_ for pPLCγ2. The Hill coefficients of the DR curves for each protein are 1.1, 2.7 and 3.3 respectively, meaning that the steepness of the DR curves increases with each downstream protein. Together, these DR parameters suggest an increased sensitivity of the response to input levels, a hallmark of the multiplicative nature of multi-step processes and positive feedback systems^22^.

Cell-to-cell variability in the response threshold can arise as a consequence of protein expression noise rather than molecular (intrinsic) noise^23,24^. The combination of the cell-to-cell response variability, as measured by the standard deviation of the half-maximal response threshold, ${\sigma }_{x50}$, and the steepness of the DR relationship, $H$, dictates the range of experimental conditions (H_2_O_2_ concentrations and exposure durations) for which bimodality can be observed. Here, the cell-to-cell variability was estimated for each protein based on the normalised median fluorescence intensity, as described by Dobrzynski et al.^25^. The variability ${\sigma }_{x50}$ is 1.04 for pCD79a, 0.59 for pSYK, and 0.71 for pPLCγ2. (Supplementary Fig. S2). Based on the estimated Hill coefficients of the DR curves and standard deviations of the threshold variability, the region for bimodality for each protein was predicted (Fig. 2b), and these regions coincide with the input concentrations for which bimodality in the population distribution was observed in the experimental data (Supplementary Fig. S1). Although pCD79a has the highest cell-to-cell variability, its shallow DR curve means that this protein generally responds more gradually to increasing H_2_O_2_ concentrations, exemplified by broad and shifting distributions; nonetheless, bimodality can be observed under specific dose-duration conditions. pSYK and pPLCγ2, on the other hand, have a more distinct switch-like response. This overall network behaviour is reminiscent of digital cell fate decision-making as the signal is propagated further downstream into the network.

To further explore the BCR signalling response and the increased sensitivity downstream in the network, we built a minimal mathematical model of the proximal BCR signalling network (Fig. 2c). The model includes phosphorylation and dephosphorylation of CD79a, SYK, PLCγ2 (sharp black arrows), and the indirect effect of H_2_O_2_ on the dephosphorylation of these proteins (flat red arrows). We include cell-to-cell variability in our model by introducing variability in the values of the Michaelis constants of each protein as well as the ${EC}_{50}$ of the phosphatases to H_2_O_2_, which are drawn from a log-normal distribution (Supplementary Fig. S3). As the model parameters are unknown for this specific cell type and cannot be uniquely estimated from the available data, we assume kinetic rates are identical for each protein (Table 1, see Methods for details). The resulting DR curves, which are constructed from 1000 simulated trajectories, qualitatively reproduce the experimental data: a decreasing half-maximal response threshold, and an increasing Hill coefficient for each more downstream protein (Fig. 2d [DR with % cells ON], Supplementary Fig. S4 [DR with median response]). Ziegler et al*.* (2019) investigated the BCR signalling response to H_2_O_2_ in CLL cells^20^. They found that the bimodal response originates from the clustering of BCRs on the membrane and a positive feedback loop from SYK onto itself. Therefore, we included a positive feedback loop on SYK in our model (Fig. 2c). However, we note that the feedback loop is not necessary to reproduce the bimodal behaviour in the model (data not shown). The DR steepness and cell-to-cell variability are sufficient to induce bimodality.

**Table 1:** Parameters used to simulate the computational model.

Parameter	Value
k_f_	1 min^−1^
k_r_	10 min^−1^
K_M1_	2 M
K_M3_	0.5 M
K_M5_	0.125 M
K_M2,_ K_M4,_ K_M6_	1 M
k_cat_	1 min^−1^
k_pos_	1 min^−1^
k_ros_	0.5 M
H	1

### Dynamic extracellular H_2_O_2_ levels reveal that the signalling response is not exclusively determined by concentration

**Figure 3:**
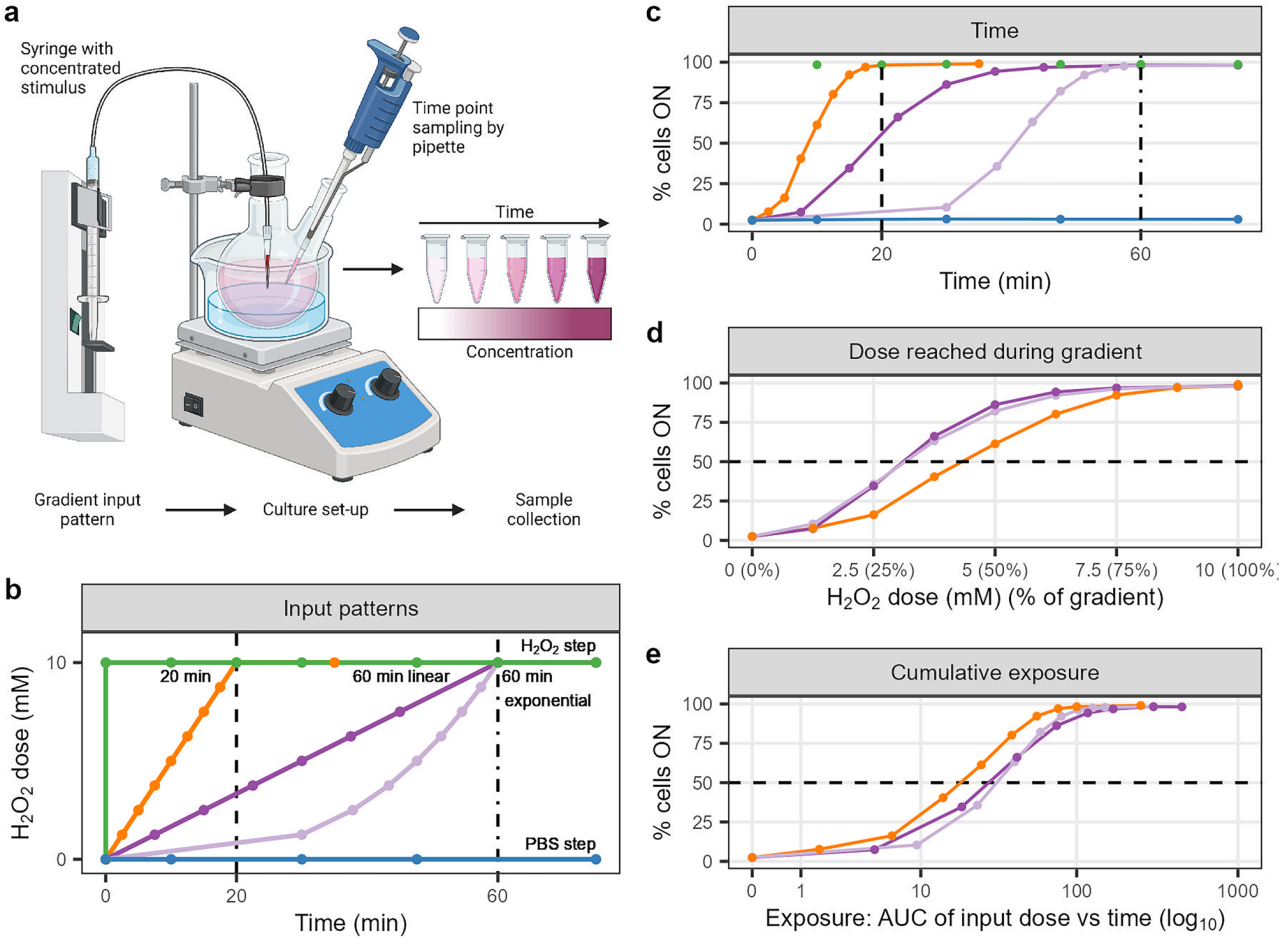
Dynamic H_2_O_2_-induced BCR signalling reveals that a combination of dose and rate of increase determine the response at intermediate doses. (**a**) Experimental set-up for creating dynamic gradient inputs. (**b**) Dynamic H_2_O_2_ input patterns. (**c**–**e**) pPLCγ2 signalling response upon gradient inputs over time (**c**), dose reached during gradients (percentage of gradient indicated) (**d**) and cumulative exposure (**e**). Blue = PBS step increase (negative control); green = H_2_O_2_ step increase (positive control); orange = 20 min linear gradient; dark purple = 60 min linear gradient; light purple = 60 min exponential gradient.

Although static step stimulations are a convenient way to study cellular signalling, dynamic environments more closely represent the in vivo microenvironment of cells and could reveal more about the signalling network capabilities. To move towards more physiologically relevant conditions, we applied temporal gradients of increasing H_2_O_2_ concentrations. The experimental set-up to create such gradients is based on Thiemicke et al*.* (2019), where a computer-controlled pump system continuously adds concentrated H_2_O_2_ to a flask with cell suspension, from which samples could be collected at various time points^26^ (Fig. 3a, see “Methods” for details). This set-up is compatible with suspension cells such as B-cells, allows coupling to phospho-specific flow cytometry as readout method, and is easy to use with standard laboratory equipment.

The initial dynamic input patterns all reached the same final concentration of 10 mM H_2_O_2_: a step increase, a linear gradient of 20 min (rate of 0.5 mM/min), a linear gradient of 60 min (rate of 0.17 mM/min), and an exponential gradient of 60 min (increasing rate during the gradient) (Fig. 3b). The signalling response of pCD79a, pSYK, and pPLCγ2 over time suggests that the response follows the input patterns of H_2_O_2_ (Fig. 3c [pPLCγ2], Supplementary Figs. S5, S6 [pCD79a + pSYK]). However, closer inspection of the response at different doses reached during the gradient reveals that the longer gradients of 60 min respond faster than the shorter gradient of 20 min, where 50% of cells ON is reached at a lower H_2_O_2_ concentration (Fig. 3d). Surprisingly, the linear and exponential gradients of 60 min respond in much the same manner even though they do not have the same rate of increase. This might be due to technical issues; the shift in response between the linear and exponential 60 min gradients might disappear in the auto-fluorescence background of the flow cytometry measurements.

To exclude the possibility that the difference in response at certain doses reached during the gradient is due to the longer exposure time of the shallower gradients, we investigated if the combination of time and dose, or the cumulative exposure, explains the signalling response (Fig. 3e). The cumulative exposure can be represented by the area under the input pattern curve (dose versus time). Although cells experiencing the 60 min gradients have a longer time to reach the same dose in the gradient, the cumulative exposure representation of the data shows that cell exposed to the steeper gradient reach a 50% response at a lower cumulative exposure, indicating that the signalling response is not solely governed by the combination of dose and time.

The model described above was used to further explore the responses to dynamic inputs. We again simulated 1000 cells with cell-to-cell variability using the parameters from Table 1. The resulting trajectories are in qualitative agreement with the experimental gradient observations (Supplementary Fig. S7), where shallow gradients are more sensitive to the concentration than steeper gradients. Noteworthy, model simulations show that early in the exponential gradient the signalling response matches that of the 60 min linear gradient since the rates of the two gradients are similar. As the rate of the exponential gradient increases and becomes more similar to that of the 20 min linear gradient, the signalling response crosses over to the response curve of the 20 min linear gradient. This behaviour is most pronounced in the simulated response of pPLCγ2. In short, both the experimental data and model simulations with dynamic input patterns reveal that the signalling response is not solely influenced by the duration, concentration, or total exposure to H_2_O_2_. Rather, the rate of increase plays a key role in determining the signalling response.

### ROS-induced signalling activation shows hallmarks of a rate-dependent sensitivity to H_2_O_2_ input concentrations

**Figure 4:**
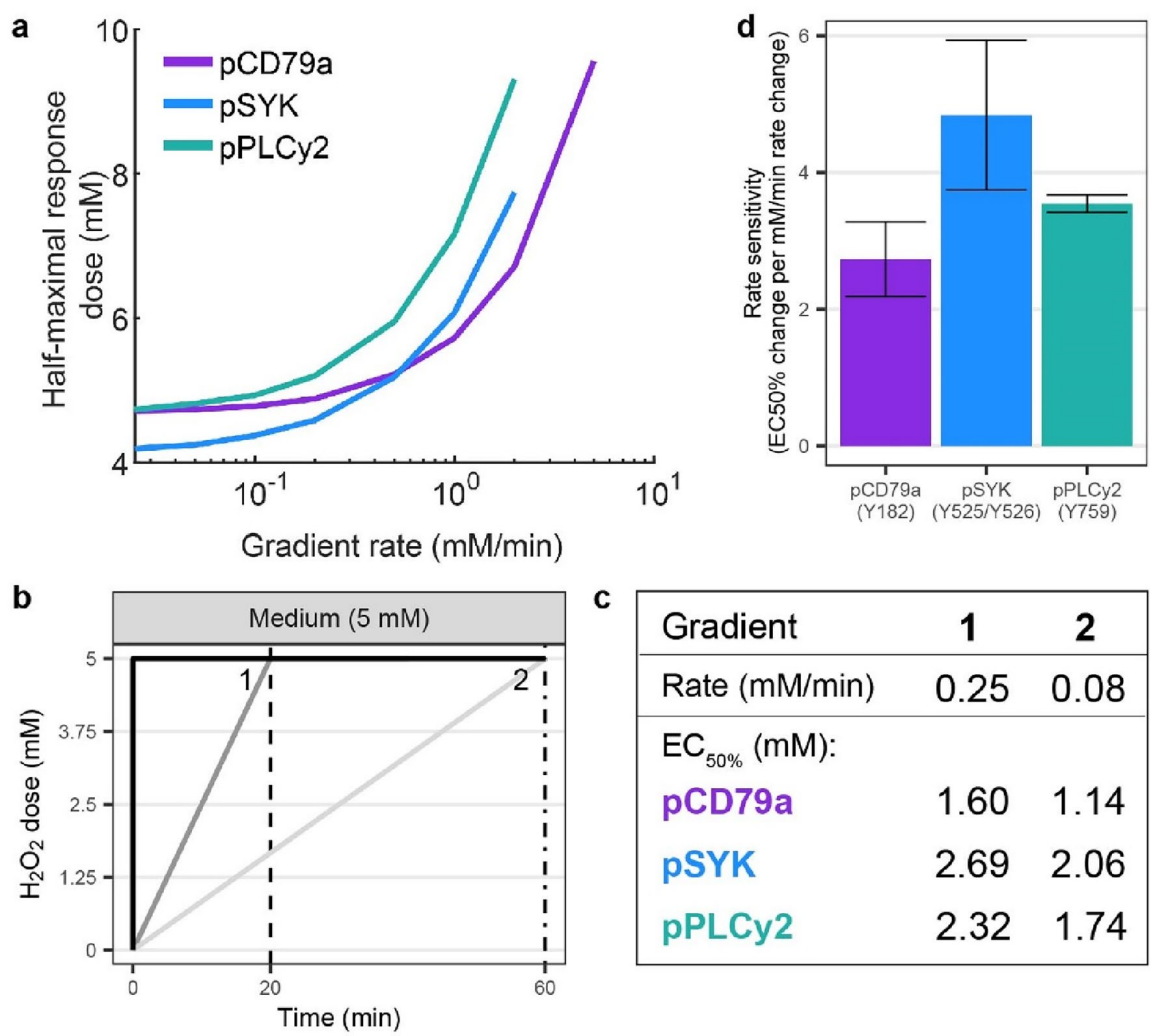
H_2_O_2_ response sensitivity can be increased by decreasing the rate of increase. (**a**) Simulated half-maximal response doses (EC_50%_) for gradients of different rates. (**b**) Two of the experimentally tested dynamic H_2_O_2_ input patterns (see Supplementary Fig. S8 for additional gradients tested). (**c**) Experimentally determined EC_50%_ for each gradient in (**b**). (**d**) Average rate sensitivity per protein (experiments n = 3), defined as the change in EC_50%_ (mM) per 1 mM/min change in rate (see Methods for details).

To comprehend more fully the influence of the rate of H_2_O_2_ increase on the signalling response, we utilised the model to simulate a wide range of gradient input rates, namely from 0.025 to 10 mM/min to a final concentration of 10 mM H_2_O_2_. For each gradient input pattern, we computed the half-maximal response dose, EC_50%_, defined as the input concentration during the gradient when 50% of cells in the population are ON (Fig. 4a). All proteins display a similar asymptotic-like curve, where an increase in the rate (steeper gradient) results in a higher EC_50%_. Thus, there is an inverse relationship between the rate of increase and the sensitivity to H_2_O_2_. This is what we also observed in the first experimental data, where the shallower gradients responded at lower doses than the steep gradient (Fig. 3d). Within the range of simulated gradients, pSYK and pPLCγ2 seem to have a higher sensitivity to changes in the rate of increase than pCD79a. This is indicated by the smaller range of rates that result in a half-maximal response concentration below 10 mM H_2_O_2_ for pSYK and pPLCγ2 (Fig. 4a x-axis), while the range of concentrations is similar for all three proteins (Fig. 4a y-axis).

Guided by these simulations, we experimentally explored a broader gradient regime (Fig. 4b, Supplementary Fig. S8a + c). To reduce the duration of experiments, gradients with the same durations but varying final concentrations were created. For each gradient, the rate and EC_50%_ were determined (Fig. 4c, Supplementary Fig. S8). These investigations show qualitative agreement with the model predictions: gradients with higher rates of increase (gradient 1 in Fig. 4b + c) have a EC_50%_ value than gradients with lower rates (gradient 2). We summarised this trend of a rate-dependent sensitivity to H_2_O_2_ into a rate sensitivity value for each protein, defined as the change in EC_50%_ per 1 mM/min change in gradient rate (averaged over 3 experiments, see Methods for details). This gives rate sensitivities of 2.73 (± 0.55 standard deviation) for pCD79a, 4.84 (± 1.09) for pSYK, and 3.55 (± 0.13) for pPLCγ2 (Fig. 4d). The highest rate sensitivity is observed for pSYK, meaning that phosphorylation of this protein is especially sensitive to the rate of H_2_O_2_ increase and occurs at lower concentrations when H_2_O_2_ levels increase slowly compared to fast increases.

Interestingly, the experimentally tested gradient condition with the lowest rate of increase, a 60 min gradient to 2.5 mM (rate of 0.04 mM/min; Supplementary Fig. 8c + d), showed no signaling activation, while the static step experiments revealed a slight response with 2.5 mM H_2_O_2_ (Fig. 1c). We postulate that the slow rate of increase in this gradient condition is too low to respond to. Since cells have a buffering capacity and can decompose H_2_O_2_^7,9^, cells might be able to remove the slowly added H_2_O_2_ and adapt to the changing environment without activating the BCR signaling network.

The concordance between the model simulations and the experimental data indicates that the behaviour results from the network topology and connections and not from specific parameters in the model. Various network motifs that elicit different responses to static and dynamic inputs have been described previously^2,4,27^. For instance, Park et al*.* (2016) found that a 3-node network motif with regulated double-negative feedback could give a transient response upon a static input, but a sustained response upon a dynamic gradient input^27^. Johnson et al*.* discovered that a rate threshold for osmotic stress signalling was governed by one of three regulatory proteins in the signalling network that were previously thought to be paralogs^2^. And Son et al*.* demonstrated that negative feedback by two proteins in a complex network enabled cells to respond to absolute differences in concentration^4^. For our cell model, the already-known network topology and connections as included in the model (Fig. 2c) are sufficient. Further research could focus on identifying which proteins or connections in the BCR network are essential for the behaviour under dynamic conditions.

## Conclusion

In short, we aimed to study B-cell signalling not only under static input conditions but also under dynamic inputs, as this is a vital difference between many in vitro studies and in vivo conditions. We found that dynamic H_2_O_2_ inputs reveal a more complex mechanism at play than static conditions suggest: A rate-dependent sensitivity to H_2_O_2_, with an increased sensitivity upon a lower rate of increase, instead of a straightforward dose-dependent response. These experimental findings could be reproduced with a minimal model of the proximal BCR signalling network. The results of this study and previous work on dynamic inputs demonstrate an enormously wide variety of mechanisms how cells can process their dynamic environment into a functional response.

### Outlook

In this study, we focussed on the fundamental mechanism behind H_2_O_2_ signalling in B-cells. Although outside the scope of this study, the biological implications of BCR activation upon ROS-induced phosphatase inhibition should be considered. Generally, low ROS levels lead to controlled growth and cellular repair, while intermediate ROS levels induce uncontrolled proliferation, and high ROS levels are toxic and induce apoptosis^12^. As the tumour microenvironment contains ROS^7^, tumour cells are exposed to these intermediate ROS levels that increase proliferation. In addition, intermediate ROS levels affect B-cell lymphomas specifically by activating intracellular signalling pathways and disrupting immune cell connections^7^. Some evidence suggests that a classic chemotherapy regimen can increase ROS levels in DLBCL and induce oxidative stress-based cytotoxicity^28^. Thus, tuning ROS levels in lymphoma cells is emerging as an intriguing new therapy approach^7,29,30^.

In addition to the biological effects of static ROS levels, the increased sensitivity to H_2_O_2_ with lower rates of increase might also have important biological effects that could be further investigated in a follow-up study. First, if H_2_O_2_ reaches tumour cells in vivo in a temporal gradient manner, a lower dose might be needed to elicit the same response as determined in static in vitro experiments. However, if the rate of H_2_O_2_ increase is too low, cells might clear the H_2_O_2_ and adapt to the changing environment, even though the total H_2_O_2_ concentration should have an effect. Second, although we focussed on the signaling responses of cells, the combination between dynamic inputs and bimodal signaling responses could result in bimodal cell fate decisions in the population where activated and non-activated cells display distinct functional responses. Third, expanding the experimental set-up to create additional types of input patterns, such as decreases in concentrations, oscillations, or randomly increasing and decreasing patterns, could give additional insights into BCR signalling under dynamic conditions. Fourth, the interplay between H_2_O_2_ and other molecules, each with their own dynamic pattern, could affect cellular responses. Since the in vivo microenvironment consists of a multitude of molecules, investigating various static or dynamic input patterns of multiple molecules would improve our understanding of in vivo signal processing. Fifth, expanding the research with additional cell models or primary cells would provide interesting comparisons, as cancer cell lines might contain (activating) mutations that alter dynamic signal processing. In short, future research efforts on ROS-induced BCR signalling should consider the dynamic nature of the in vivo tumour microenvironment in order to elucidate B-cell signalling under dynamic conditions and what this means for disease development, progression, and treatment.

## Materials and methods

### Cell culture

The HBL1 cell line (gift from Prof. Dr. Annemiek van Spriel, Radboud University Medical Center) is an EBV-negative ABC-DLBCL cell line (see Table 2 for all materials and reagent information). It originates from a Japanese 65-year-old male and was established in 1988^31^. Cells were cultured in RPMI 1640 medium supplemented with 10% (v/v) fetal bovine serum (FBS) and 1% (v/v) penicillin/streptomycin. Cells were maintained at a density of 0.20–1.5*10^6^ cells/mL in a humidified incubator at 37 °C with 5% CO_2_.

**Table 2:** Key resources table.

Reagent or resource	Source	Identifier
Antibodies		
Caspase 3: V450 Rabbit Anti-Active Caspase-3 (1/500)	BD Biosciences	Cat#560,627; RRID:AB_1727415; clone:C92-605
PARP: BV421 Mouse Anti-Cleaved PARP (Asp 214) (1/500)	BD Biosciences	Cat#564,129; RRID:AB_2738611; clone:F21-852
pSYK: Phospho-Syk (Tyr525/526) (C87C1) Rabbit mAb (PE Conjugate) (1/250)	Cell Signalling Technologies	Cat#6485; RRID:AB_11220429; clone:C87C1
pPLCγ2: Alexa Fluor® 647 Mouse anti-PLC-γ2 (pY759) (1/100)	BD Biosciences	Cat#558,498; RRID:AB_647139; clone:K86-689.37
pCD79a: Phospho-CD79A (Tyr182) (D1B9) Rabbit mAb (Alexa Fluor® 488 Conjugate) (1/250)	Cell Signalling Technologies	Cat#52,821; RRID:AB_2799422; clone:D1B9
Chemicals, peptides, and recombinant proteins		
H_2_O_2_	ThermoFisher	Cat#202,460,010
RPMI Medium 1640	Gibco	Cat#52,400–025
FBS	Gibco	Cat#A3160801
Penicillin/streptomycin	Gibco	Cat#15,140–122
PFA	Merck	Cat#1,040,051,000
Nuclease-free water	Ambion	Cat#AM9937
Tris–HCl, pH 7.5	Gibco	Cat#15,567–027
NaCl	Sigma Aaldrich	Cat#S5886
Triton X-100	Thermo Fisher Scientific	Cat#85,112
Dulbecco's phosphate buffered saline	Gibco	Cat#14,190–094
0.5 M EDTA, pH 8.0	Lonza	Cat#51,201
BSA	Sigma Aaldrich	Cat#A4503-50G
HFE-7500 3 M (TM) Novec (TM) Engineered fluid	Fluorochem	Cat#051,243
Experimental models: Cell lines		
Human: HBL1	Gift from Prof. Dr. Annemiek van Spriel (Tumour Immunology, RadboudUMC)	RRID:CVCL_4213
Software and algorithms		
neMESYS UserInterface version 2016.6.14.1	Cetoni	NA
BD FACSuite software v1.0.6	BD Biosciences	NA
R version 4.0.5	The R Foundation	https://www.r-project.org/
RStudio	RStudio, USA	https://www.rstudio.com/
BioRender	BioRender, Canada	https://biorender.com/
Other		
NeMESYS pump system	Cetoni	NA
Fine dosage syringe	Braun	Cat#9161406 V
HENKE-JECT hypodermic needle 0.6 × 25 mm	VWR	613–2017
PTFE tubing 0.81 mm DD × 1.63 mm OD	adtech	Cat#77,228

### Plate-based step stimulation procedure

Prior to experiments, cells were counted, centrifuged for 5 min at 300 rcf, resuspended at 5.3*10^6^ cells/mL in serum-poor medium (RPMI 1640 medium with 2% FBS and 1% pen/strep) and rested for at least 60 min at 37 °C. 0.8*10^6^ cells per well were distributed in 96-well plates. Working backwards from the longest stimulation time, H_2_O_2_ was added to the wells (final concentrations of 0, 0.25, 1, 2.5, 5, 10 or 20 mM) at desired time points and cells were incubated at 37 °C with 5% CO_2_. All samples were fixated simultaneously by adding 4% final concentration paraformaldehyde (PFA) for 15 min at room temperature (RT). The fixation was stopped by centrifuging samples for 5 min at 800 rcf and resuspending them in phosphate buffered saline (PBS). Samples were either immediately processed (see phospho-specific flow cytometry of fixated cells) or stored overnight at 4 °C before further processing.

### Flask-based step and gradient stimulation set-up and procedure

The experimental set-up consists of a round-bottom 2-neck flask held in a small water bath set on a hot plate stirrer (37 °C, 500 rpm), a computer-controlled pump system, and a syringe with needle and tubing. Prior to experiments, gradient pump profiles were generated for all gradient conditions via a custom R script using the following parameters: initial reaction volume (volume needed for all samples + 5 mL extra), stimulus concentration, desired final concentration, gradient duration, gradient pattern (linear or exponential), sample volume, and sample time points. The exponential gradient of 60 min to 10 mM H_2_O_2_ is described with the following formula:

1 $$\begin{array}{c}concentration= \frac{60}{{10}^\frac{1}{3}} *{time}^\frac{1}{3}.\end{array}$$

The generated pump profiles were uploaded to the pump system. A syringe with a needle attached was filled with HFE-7500 oil. Then, tubing and a sterile pipette tip were attached, connected by a polydimethylsiloxane (PDMS) stopper. The required amount of concentrated H_2_O_2_ stimulus was loaded into the oil-filled pipette tip, thereby reducing waste of stimulus.

Prior to experiments, cells were counted, centrifuged for 5 min at 300 rcf, resuspended at 2*10^6^ cells/mL in serum-poor medium and rested for at least 60 min at 37 °C. Rested cells were placed in the flask, the stimulus-loaded pipette tip was inserted, and all flask openings were covered with tape or parafilm. For step stimulations, the stimulus (H_2_O_2_ or PBS) was added manually by pipette. Samples of 0.5–1*10^6^ cells were collected manually by pipette and immediately fixated with 4% final concentration PFA for 15 min at RT. The fixation was stopped by centrifuging samples for 5 min at 800 rcf and resuspending them in PBS. Samples were either immediately processed further (see phospho-specific flow cytometry of fixated cells) or stored overnight at 4 °C before further processing.

Sample moments varied between experiments. During gradient stimulations, samples were taken when certain concentrations were reached in the gradient profile, namely at 0, 1.25, 2.5, 3.75, 5, 6.25, 7.5 8.75, and 10 mM H_2_O_2_. The step stimulation samples matched a time point that a sample was taken in a gradient stimulation in the same experiment.

### Phospho-specific flow cytometry of fixated cells

PFA-fixed cells were transferred to 96-well plates (0.25–0.5*10^6^ cells/well), for multiple staining panels samples were split across multiple wells. After 5 min centrifugation at 800 rcf, cells were permeabilised for 10 min in NF water with 100 mM Tris–HCl, 150 mM NaCl and 0.1% Triton X-100 at RT. Cells were then washed 1 × with FACS buffer (Dulbecco’s PBS with 0.1% bovine serum albumin, 0.05% sodium azide and 0.5 mM EDTA) before resuspending in 40 µL staining solution, consisting of fluorescently labelled antibodies in FACS buffer (see Table 3 for antibody combinations), and incubated for 1 h at RT in the dark. Cells were washed 3 × with FACS buffer and finally resuspended in FACS buffer for measurements. Cells were measured on the BD FACSVerse (BD Biosciences) (50,000 events per sample). Compensation matrices were created from single-stain controls of each antibody used and applied to all measurements.

**Table 3:** Antibody panels for phospho-specific flow cytometry.

Target	Fluorophore	Dilution
Panel A		
Cleaved Caspase 3	V450	1/500
Cleaved PARP	BV421	1/500
pSYK	PE	1/200
pPLCγ2	Alexa 647	1/100
pCD79a	Alexa 488	1/200
Panel B		
Cleaved Caspase 3	V450	1/500
Cleaved PARP	BV421	1/500
Rabbit IgG isotype	Alexa 647	1/1000
Panel C		
Buffer (no stain)	NA	NA

*NA* not applicable.

### Flow cytometry data analysis

All flow cytometry data were analysed in RStudio with various packages (see session information of the data analysis files for all packages and version numbers). The flow cytometry data was gated using the flowCore^32^ and ggcyto^33^ packages. The data were first gated to remove debris (FSC-A vs SSC-A), then to select singlets (FSC-H vs FSC-W), and then to select live cells (FSC-A vs active caspase 3 + cleaved PARP, BV421-A) (Supplementary Fig. S9). The gated flow cytometry data were then processed and visualised using the tidyverse package^34^. Samples with < 5000 gated cells were removed from the analysis. Median fluorescent intensities (MFI) for each fluorophore in each sample were calculated, as well as the percentage of activated cells (percentage ON), where the ON threshold is the fluorescence at which 97.5% of cells at time = 0 min or concentration = 0 mM H_2_O_2_ fall below this value (for each condition). For gradient data, the rate sensitivity, defined as the change in half-maximal response dose per 1 mM/min change in the rate, was calculated for each experiment separately as follows:

2 $$\begin{array}{c}Rate \,sensitivity= \frac{{dose}_{50}^{steep}- {dose}_{50}^{shallow}}{{rate}^{steep}- {rate}^{shallow}},\end{array}$$

where ${dose}_{50}$ is the concentration during the gradient when 50% cells ON is reached, steep indicates the steepest gradient of the experiment and shallow indicates the shallowest gradient of the same experiment.

### Dose response curves

Dose response curves from flow cytometry data were fit on (1) the fold change of the normalised MFI and (2) the percentage ON. For both cases, the unstimulated sample at the time point of interest for each replicate is taken as the control. The dose response is assumed to follow Hill-type kinetics:

3 $$\begin{array}{c}R={R}_{0}+{R}_{max}\frac{{S}^{H}}{{S}^{H}+E{C}_{50}^{H}},\end{array}$$

where ${R}_{0}$ is the response level in the absence of stimulus $S$, ${R}_{max}$ is the response amplitude, $E{C}_{50}$ is the half-maximal response threshold, and $H$ is the Hill coefficient.

### Population-level bimodality from the interplay of nonlinearity in input–output and cell-to-cell variability

Dobrzynski et al*.* have analytically quantified conditions for the existence of bimodality in the presence of response threshold variability^25^. We have applied their mathematical framework to our data to infer the level of threshold variability ${\sigma }_{x50}$ for each protein. If this parameter, together with the Hill coefficient of the DR curve, satisfies the condition $H\cdot {\sigma }_{x50}>\surd 2$, bimodal distribution may emerge. Our analysis revealed that the necessary condition for bimodality was satisfied for pSYK and pPLCγ2, but not for pCD79a. Furthermore, we can prospect the range of stimulus concentration for which we can expect to see a bimodal population response, which is also solely determined by ${\sigma }_{x50}$ and the steepness $H$ of the DR curve. For further details on the analytical method, we refer to the source paper by Dobrzynski et al. (2014).

### Computational model of the phosphorylation cascade

We model each protein as a switch, in which a substrate is phosphorylated by a kinase. The phosphorylation can subsequently be reversed by a phosphatase. ROS such as H_2_O_2_ enhance signalling by oxidising ROS-sensitive signalling molecules such as phosphatases^17^. This oxidation broadly inactivates tyrosine phosphatases, which can be modelled by a Hill-type inhibition. We model the effective concentration of active phosphatase $P$ as a function of H_2_O_2_ by

4 $$\begin{array}{c}P=\frac{1}{1+{\left(\frac{S}{{k}_{ros}}\right)}^{H}},\end{array}$$

where $P$ is the (scaled) concentration of active phosphatase, ${k}_{ros}$ is the half-maximal concentration constant, $H$ is the Hill coefficient and $S$ refers to the H_2_O_2_ concentration.

The concentration of an isolated phosphorylated protein can be described by the following ordinary differential equation (ODE):

5 $$\begin{array}{c}\frac{d{A}_{p}}{dt}={k}_{f}\frac{{A}_{T}-{A}_{p}}{{K}_{{M}_{1}}+{\left({A}_{T}-{A}_{p}\right)}}-{k}_{r}P\frac{{A}_{p}}{{K}_{{M}_{2}}+{A}_{p}} ,\end{array}$$

where ${A}_{T}$ is the total protein concentration, ${A}_{p}$ is the concentration of phosphorylated protein, ${k}_{f}$ is the phosphorylation rate, ${k}_{r}$ is the dephosphorylation rate, and ${K}_{{M}_{i}}$ refers to the Michaelis constant. Michaelis–Menten rate laws were chosen to model the underlying protein dynamics as the work of Dobrzynski et al*.* state the presence of a sigmoidal dose–response relationship as a condition for population bimodality^25^.

Our simplified model includes all experimentally measured proteins which form a phosphorylation cascade. In such a cascade, each phosphorylated protein acts as a kinase that phosphorylates the next protein in the cascade. Additionally, it has been shown that SYK acts as a positive feedback regulator on the BCR-associated tyrosine kinases LYN (not included in our model) and SYK itself^20^. Therefore, we include this positive autoregulation in our model. The system of ODEs describing the simplified phosphorylation cascade is then given by:

6 $$\begin{array}{c}\frac{d{X}_{p}}{dt}={k}_{f}\frac{{A}_{T}-{A}_{p}}{{K}_{{M}_{1}}+{\left({A}_{T}-{A}_{p}\right)}}-{k}_{r}P\frac{{A}_{p}}{{K}_{{M}_{2}}+{A}_{p}}\end{array}$$

7 $$\begin{array}{c}\frac{d{Y}_{p}}{dt}={k}_{f}{X}_{p}\frac{{Y}_{T}-{Y}_{p}}{{K}_{{M}_{3}}+{\left({Y}_{T}-{Y}_{p}\right)}}+{k}_{cat}\frac{{Y}_{T}-{Y}_{p}}{{k}_{pos}+{\left({Y}_{T}-{Y}_{p}\right)}}-{k}_{r}P\frac{{Y}_{p}}{{K}_{{M}_{4}}+{Y}_{p}}\end{array}$$

8 $$\begin{array}{c}\frac{d{Z}_{p}}{dt}={k}_{f}{Y}_{p}\frac{{Z}_{T}-{Z}_{p}}{{K}_{{M}_{5}}+{\left({Z}_{T}-{Z}_{p}\right)}}-{k}_{r}P\frac{{Z}_{p}}{{K}_{{M}_{6}}+{Z}_{p}},\end{array}$$

where we denote pCD79a by $X$, pSYK by $Y$, and pPLCγ2 by $Z$. Using MATLAB, we resolved this system of equations using the built-in function ode23. All protein abundances are scaled between 0 and 1 and are initially (in the absence of stimulus) assumed to be in an unphosphorylated state. As the values of the model parameters are unknown for the cell line used in our experiments, and due to the increased number of parameters in equations with Michaelis–Menten kinetics as compared to linear mass-action kinetics, we simulated the model using a general parameter set (Table 1). Here, we have assumed that the Michaelis constants ${K}_{{M}_{j}}, j\in \{\mathrm{1,3},5\}$ become successively smaller as we move further down the cascade, to reflect sensitivity propagation and amplification of downstream proteins in signaling cascades^35–37^. All other parameters are assumed to be equal for each protein.

In addition to stationary H_2_O_2_ levels, we extend the ODE model to include time-dependent inputs by including a differential equation that describes the gradient profile (linear of exponential). This equation is characterised by two parameters, namely the gradient duration and maximal concentration. Here, we have assumed degradation of H_2_O_2_ is negligible on the time scale of our experiments.

We model cell-to-cell variability in the population response by generating variation in the thresholds ${K}_{{M}_{i}}, i\in \{\mathrm{1,2},\mathrm{3,4},\mathrm{5,6}\}$ and ${k}_{ros}$. Variation in the values of these parameters can reflect variability in the levels of signalling components, such as kinase and phosphatase levels. We generate 1000 cells with ${K}_{M}$ and ${k}_{ros}$ values randomly chosen from a lognormal distribution with mean ${m}_{x}$ and variance ${m}_{x50}\cdot {s}_{x50}^{2}$. We used ${m}_{x50}$ as given by the values in Table 1, and ${s}_{x50}=1.25$ in all simulations to reflect large cell-to-cell variability and found that this was sufficient to generate bimodal responses in the levels of ${Y}_{p}$ and ${Z}_{p}$, but not ${X}_{p}$, in agreement with experimental observations. To assess the percentage of activated cells (% cells ON), cells are said to be activated if its protein abundance exceeds 30% of its maximal level. The half-maximal response concentration as given in Fig. 4a is calculated as the H_2_O_2_ concentration at which 50% of cells are activated. At higher gradient rates, the gradients become very short so 50% is not reached, resulting in a discontinuation of the line.

### Supplementary Information

Supplementary Figures.

## Data Availability

Data analysis was performed using custom R scripts (flow cytometry data analysis), Python scripts (threshold variability analysis), and MATLAB scripts (BCR model simulations). Together with the raw flow cytometry data, these are publicly available as of the date of publication at https://github.com/huckgroup/H2O2_manuscript. Any additional information required to analyse the data reported in this paper is available from the corresponding author upon request.

## References

[1] Heemskerk I (2019). Rapid changes in morphogen concentration control self-organized patterning in human embryonic stem cells. Elife.

[2] Johnson AN (2021). A rate threshold mechanism regulates MAPK stress signaling and survival. Proc. Natl. Acad. Sci. U. S. A..

[3] O'Donoghue GP (2021). T cells selectively filter oscillatory signals on the minutes timescale. Proc. Natl. Acad. Sci. U. S. A..

[4] Son MJ (2021). NF-kappa B responds to absolute differences in cytokine concentrations. Sci. Signal..

[5] Thiemicke A, Neuert G (2023). Rate thresholds in cell signaling have functional and phenotypic consequences in non-linear time-dependent environments. Front. Cell Dev. Biol..

[6] Thiemicke A, Neuert G (2021). Kinetics of osmotic stress regulate a cell fate switch of cell survival. Sci. Adv..

[7] Domka K, Goral A, Firczuk M (2020). cROSsing the line: Between beneficial and harmful effects of reactive oxygen species in B-cell malignancies. Front. Immunol..

[8] Reth M (2002). Hydrogen peroxide as second messenger in lymphocyte activation. Nat. Immunol..

[9] Zhang H, Wang L, Chu Y (2019). Reactive oxygen species: The signal regulator of B cell. Free Radic. Biol. Med..

[10] Kirtonia A, Sethi G, Garg M (2020). The multifaceted role of reactive oxygen species in tumorigenesis. Cell. Mol. Life Sci..

[11] Kusowska A (2022). Molecular aspects of resistance to immunotherapies-advances in understanding and management of diffuse large B-cell lymphoma. Int. J. Mol. Sci..

[12] Moloney JN, Cotter TG (2018). ROS signalling in the biology of cancer. Semin. Cell Dev. Biol..

[13] Niemann CU, Wiestner A (2013). B-cell receptor signaling as a driver of lymphoma development and evolution. Semin. Cancer Biol..

[14] Tanaka S, Baba Y, Wang JY (2020). B cell receptor signaling. Advances in Experimental Medicine and Biology.

[15] Feng YY (2019). Essential role of NADPH oxidase-dependent production of reactive oxygen species in maintenance of sustained B cell receptor signaling and B cell proliferation. J. Immunol..

[16] Wheeler ML, Defranco AL (2012). Prolonged production of reactive oxygen species in response to B cell receptor stimulation promotes B cell activation and proliferation. J. Immunol..

[17] Tsubata T, Wang JY (2020). Involvement of reactive oxygen species (ROS) in BCR signaling as a second messenger. Advances in Experimental Medicine and Biology.

[18] Irish JM, Czerwinski DK, Nolan GP, Levy R (2006). Kinetics of B cell receptor signaling in human B cell subsets mapped by phosphospecific flow cytometry. J. Immunol..

[19] Palazzo AL (2011). Association of reactive oxygen species-mediated signal transduction with in vitro apoptosis sensitivity in chronic lymphocytic leukemia B cells. PLoS One.

[20] Ziegler CGK (2019). Constitutive activation of the B cell receptor underlies dysfunctional signaling in chronic lymphocytic leukemia. Cell Rep..

[21] Polikowsky HG, Wogsland CE, Diggins KE, Huse K, Irish JM (2015). Cutting edge: Redox signaling hypersensitivity distinguishes human germinal center B cells. J. Immunol..

[22] Frank SA (2013). Input-output relations in biological systems: Measurement, information and the Hill equation. Biol. Direct..

[23] Birtwistle MR (2012). Emergence of bimodal cell population responses from the interplay between analog single-cell signaling and protein expression noise. BMC Syst. Biol..

[24] Topolewski P (2022). Phenotypic variability, not noise, accounts for most of the cell-to-cell heterogeneity in IFN-gamma and oncostatin M signaling responses. Sci. Signal..

[25] Dobrzynski M (2014). Nonlinear signalling networks and cell-to-cell variability transform external signals into broadly distributed or bimodal responses. J. R. Soc. Interface.

[26] Thiemicke A, Jashnsaz H, Li G, Neuert G (2019). Generating kinetic environments to study dynamic cellular processes in single cells. Sci. Rep..

[27] Park SM, Shin SY, Cho KH (2016). A regulated double-negative feedback decodes the temporal gradient of input stimulation in a cell signaling network. PLoS One.

[28] Mai Y (2016). An oxidative stress-based mechanism of doxorubicin cytotoxicity suggests new therapeutic strategies in ABC-DLBCL. Blood..

[29] Fidyt K (2019). Targeting the thioredoxin system as a novel strategy against B-cell acute lymphoblastic leukemia. Mol. Oncol..

[30] Yosifov DY (2020). Oxidative stress as candidate therapeutic target to overcome microenvironmental protection of CLL. Leukemia.

[31] Nozawa Y (1988). Establishment and characterization of an epstein-barr virus negative B-cell lymphoma cell-line and successful heterotransplantation. Tohoku J. Exp. Med..

[32] Hahne F (2009). flowCore: A bioconductor package for high throughput flow cytometry. BMC Bioinform..

[33] Van P, Jiang W, Gottardo R, Finak G (2018). ggCyto: Next generation open-source visualization software for cytometry. Bioinformatics.

[34] Wickham H (2019). Welcome to the Tidyverse. J. Open Source Softw..

[35] Ferrell JE (1997). How responses get more switch-like as you move down a protein kinase cascade. Trends Biochem. Sci..

[36] Klipp E, Liebermeister W (2006). Mathematical modeling of intracellular signaling pathways. BMC Neurosci..

[37] Rácz É, Slepchenko BM (2008). On sensitivity amplification in intracellular signaling cascades. Phys. Biol..

