# Supplementary material for: Dynamic hydrogen peroxide levels reveal a rate-dependent sensitivity in B-cell lymphoma signaling
Source: Sci Rep. 2024 Feb 21;14:4265. doi: 10.1038/s41598-024-54871-7 (PMC10882005; doi:10.1038/s41598-024-54871-7)
Supplement: Supplementary file 1 — Supplementary Figures. [file 41598_2024_54871_MOESM1_ESM.docx]

Dynamic hydrogen peroxide levels reveal a rate-

dependent sensitivity in B-cell lymphoma signaling

Melde Witmond^1^, Emma Keizer^1^, Bas Kiffen^1^, Wilhelm Huck^1,^*, Jessie van Buggenum^1,2,^*

^1^ Institute of Molecules and Materials (IMM), Radboud University Nijmegen, the Netherlands

^2^ Single Cell Discoveries (SCD), Utrecht, the Netherlands

* Shared corresponding author

Corresponding authors: Wilhelm Huck (email [wilhelm.huck@ru.nl](mailto:wilhelm.huck@ru.nl)); Jessie van Buggenum (email: [jessie.vanbuggenum@ru.nl](mailto:jessie.vanbuggenum@ru.nl))

# Supplementary data


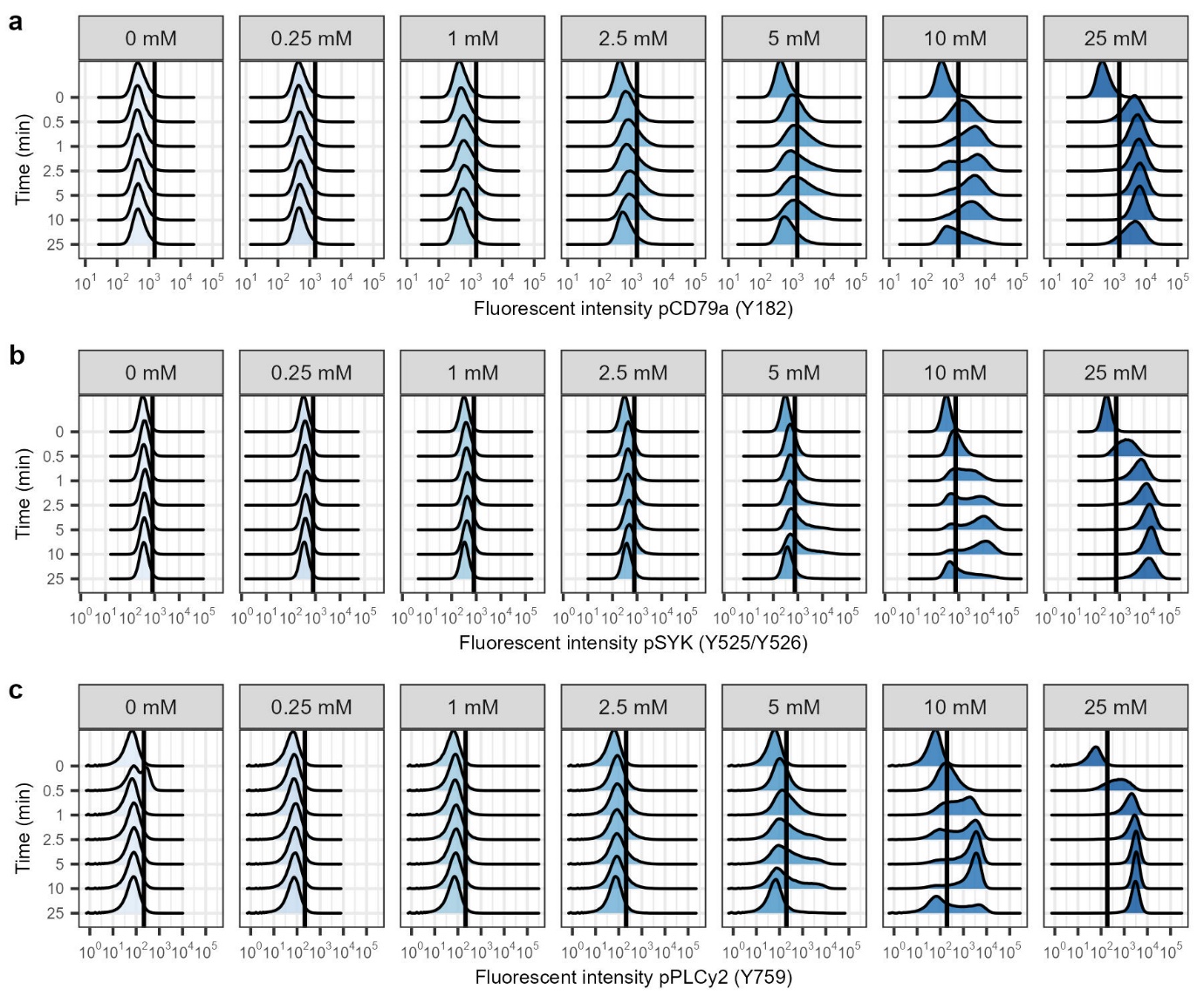


**Supplementary Figure S1: Static step stimulation with H_2_O_2_, all concentrations and time points used for Figure 1.** a) pCD79a. b) pSYK. c) pPLCγ2. Colour indicates H_2_O_2_ concentration.


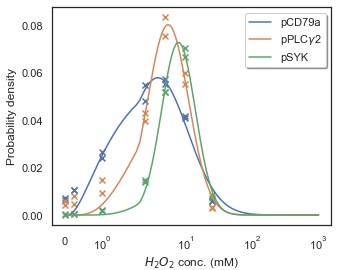


**Supplementary Figure S2: Estimation of cell-to-cell variability in signaling response upon static H_2_O_2_ stimulation.** Based on experimental data at t=10 min stimulation. Method: Dobrzynski et al. (2014) (see Methods section).


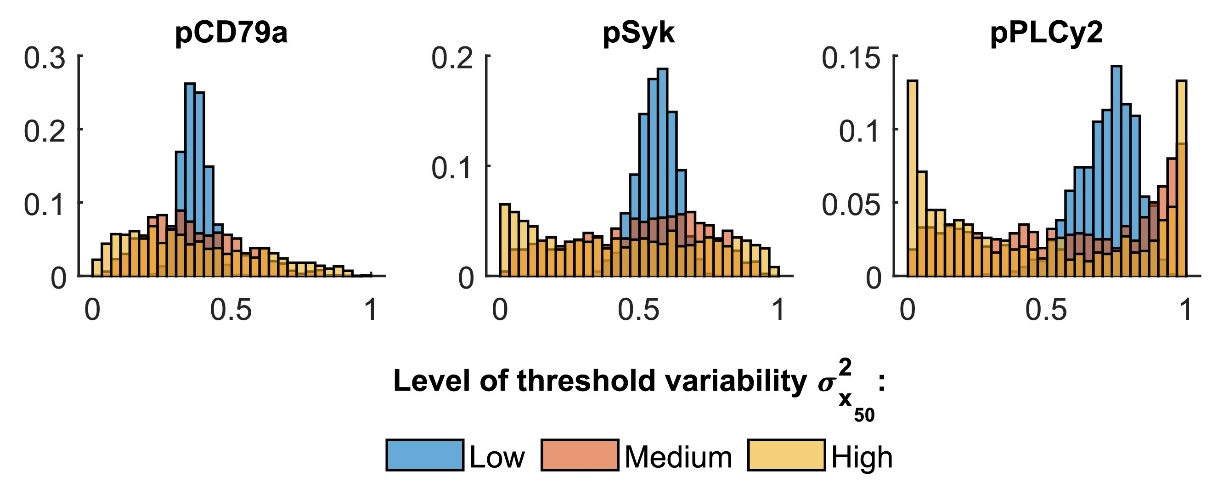


**Supplementary Figure S3: Effect of low, medium, or high cell-to-cell variability in the model on simulated signaling response.**


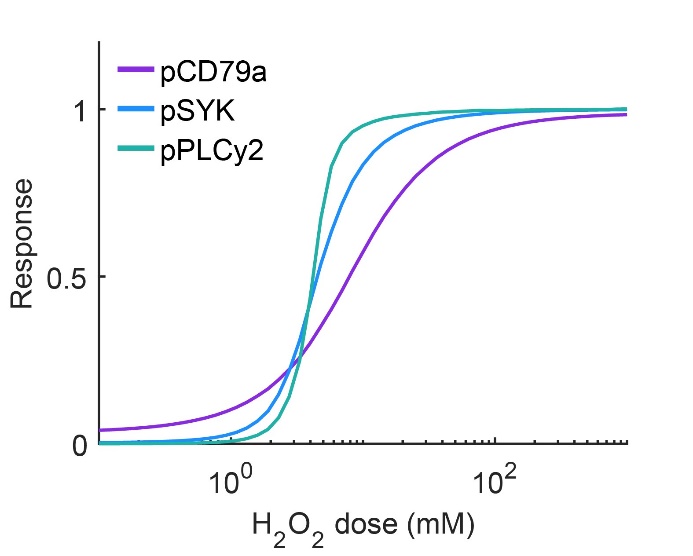


**Supplementary Figure S4: Dose response curves based on the median response, simulated via the minimal model.**


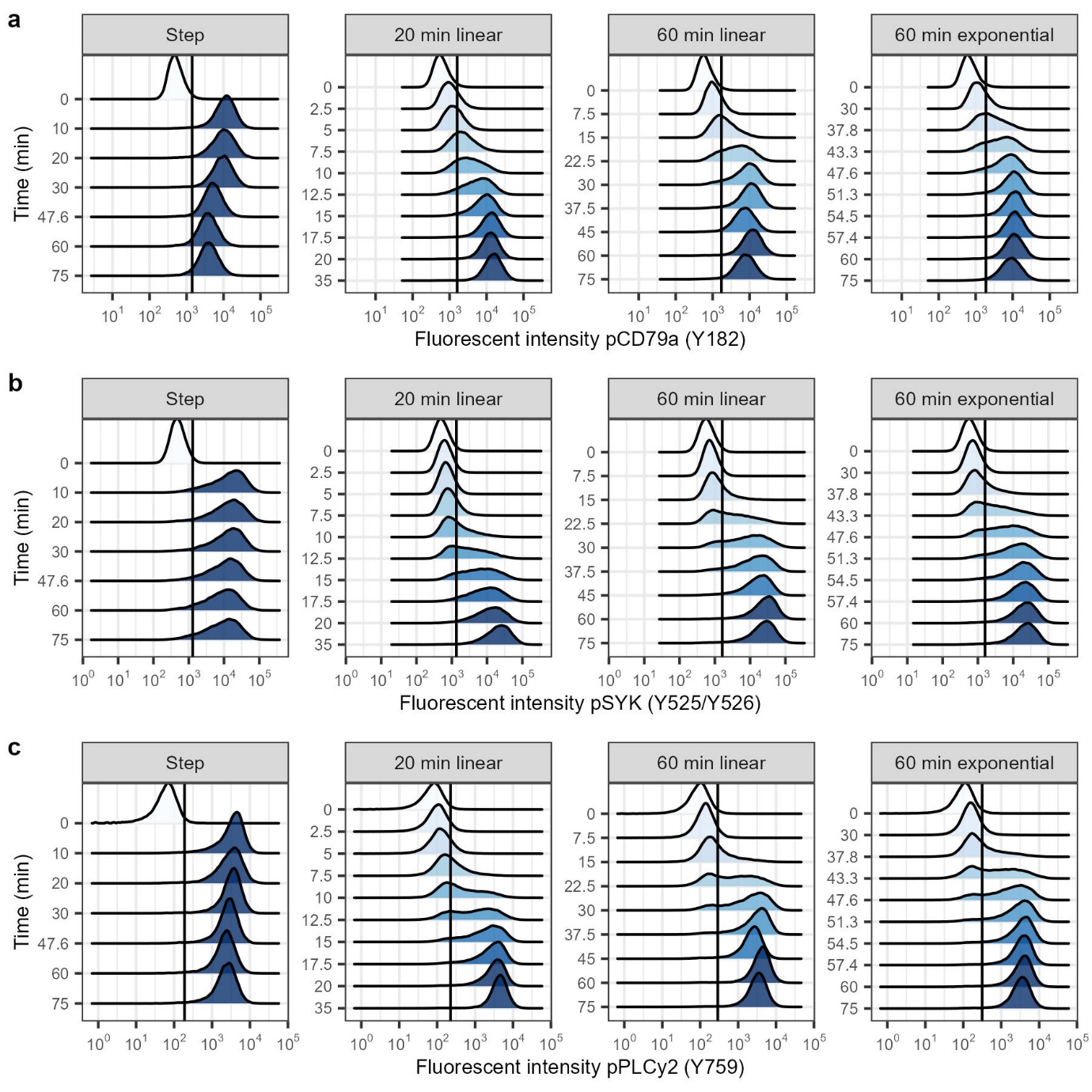


**Supplementary Figure S5: Dynamic stimulation with a step, 20 min linear gradient, 60 min linear gradient, and 60 exponential gradient of H_2_O_2_ as input patterns.** a) pCD79a. b) pSYK. c) pPLCγ2. Colour indicates H_2_O_2_ concentration.


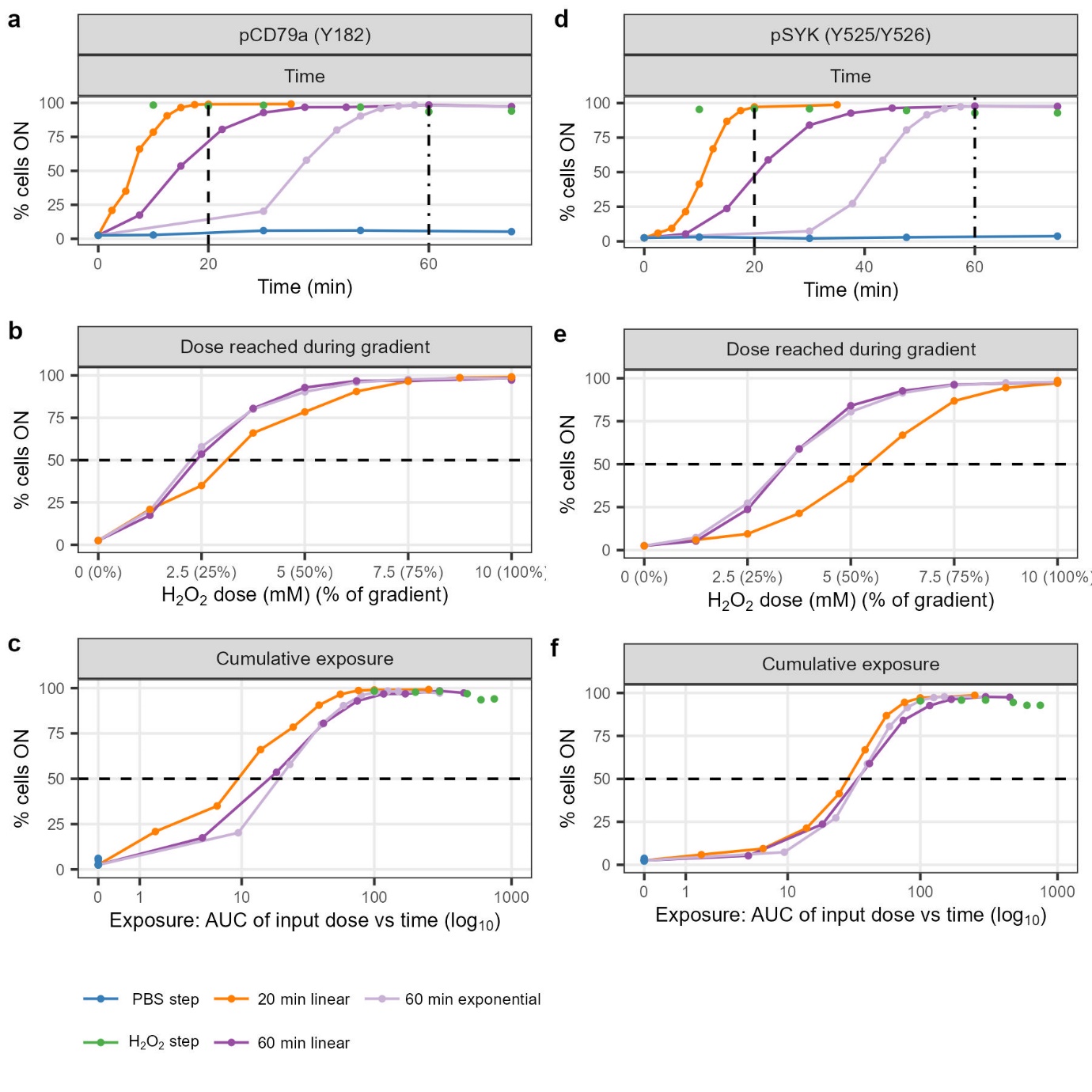


**Supplementary Figure S6: Signaling response to dynamic inputs.** a-c) pCD79a response plotted against time (a), H_2_O_2_ dose reached during the gradient (b) and cumulative exposure (c). d-e) pSYK plotted against time (d), H_2_O_2_ dose reached during the gradient (e) and cumulative exposure (f).


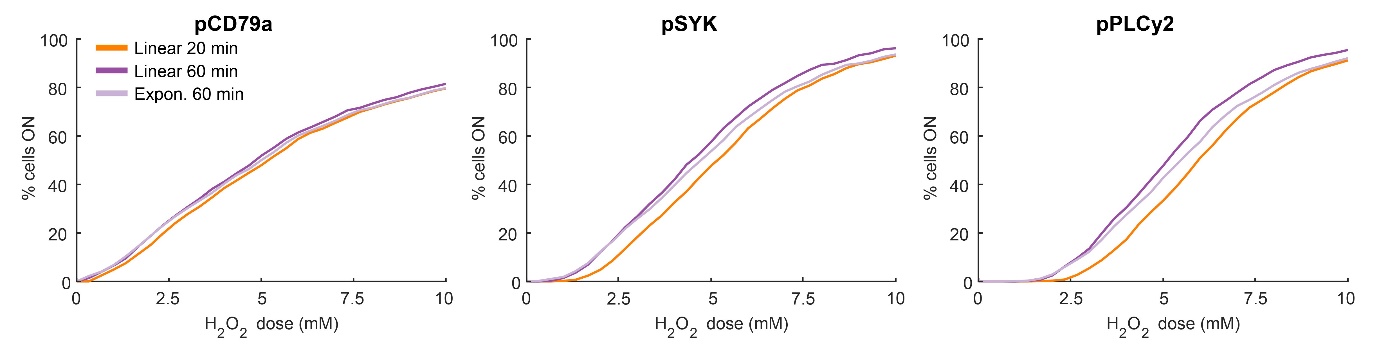


**Supplementary Figure S7: Simulated signaling response upon dynamic inputs at different concentrations during the gradient.**

**
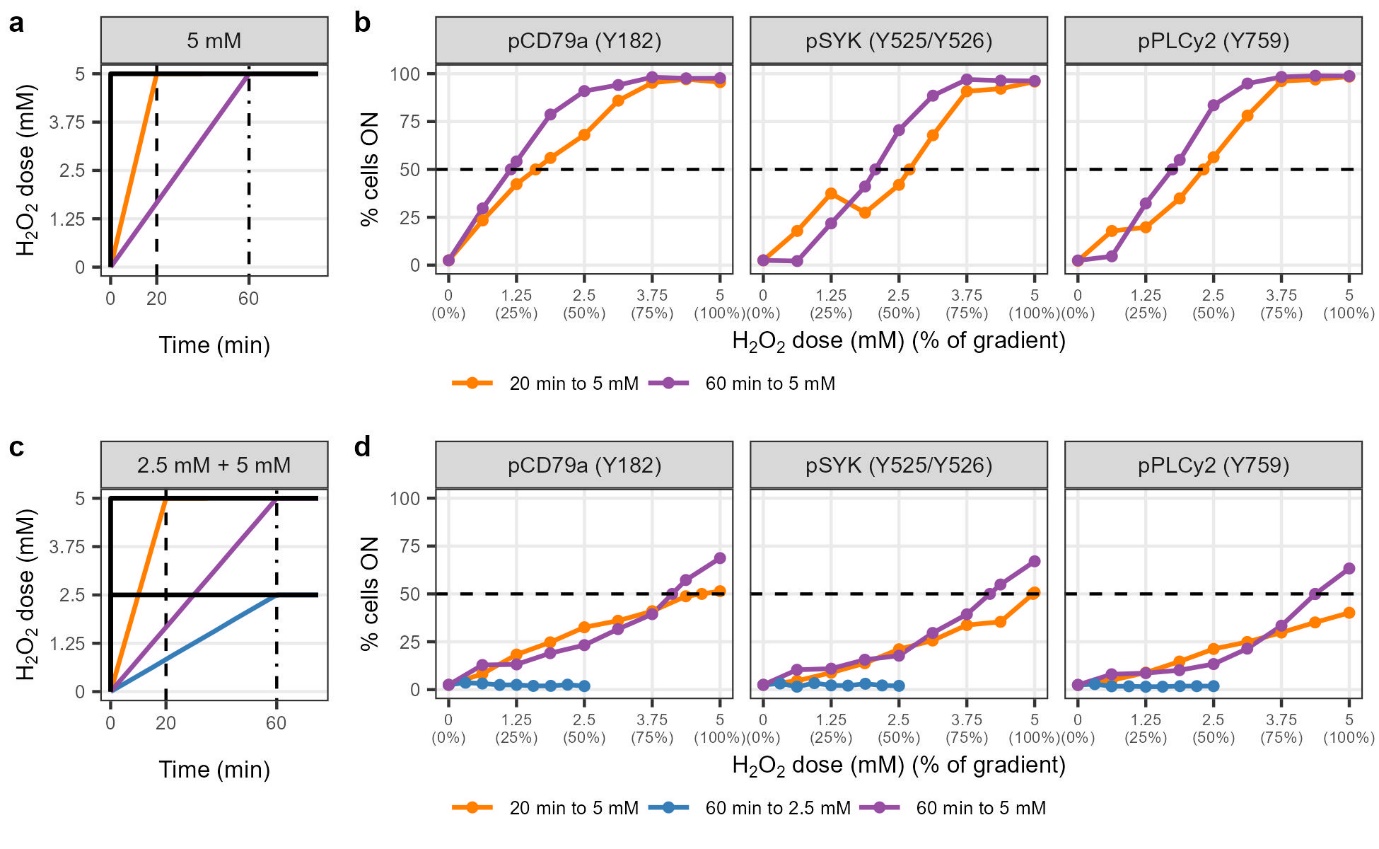
**

**Supplementary Figure S8: Signaling response of various experimentally tested gradient conditions.** a+c) Dynamic input patterns during two different experiments. b+d) Signaling response at different doses during the gradient stimulations of a+c. The EC_50_ is indicated with a dashed line. c) Data behind Figure 4c.


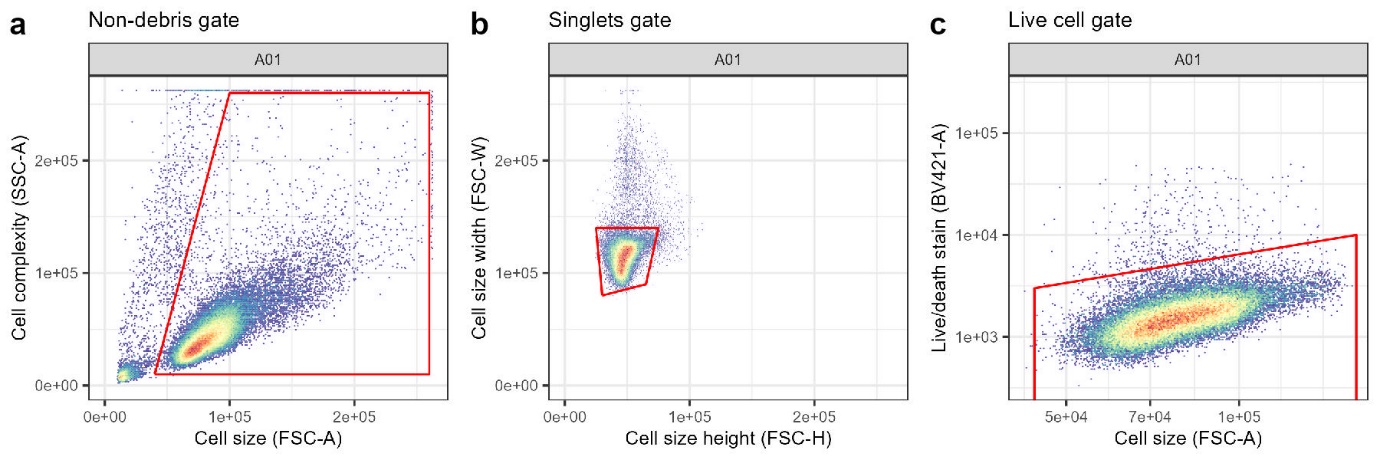


**Supplementary Figure S9: Gating strategy for an exemplary sample (from the static conditions experiment).** a) Gating on debris. b) Gating on single cells. c) Gating on live cells.
